# Nervousness, Debilities, Etc.

**Published:** 1862-08

**Authors:** 


### NERVOUSNESS, DEBILITIES, ETC.

The nervous fluid is manufactured from the blood; the nerves themselves are nourished and repaired by the blood. The whole nervous system may become diseased in three ways: first, by sudden shocks; second, by excessive action; third, by an unnatural condition of the blood for a long time. That mental shocks, as from fear, or bad news, may prostrate the nervous system, destroy the mind, and life itself, needs no argument. That hard work, insufficient sleep, too great a press of business, or too much time spent in study, with errors in eating and neglect of exercise, may impair the nervous system, by calling the nervous fluid into action or use, before it is fully "ripe" or matured, is an often observed fact. The remedy, and the only remedy for these is, an avoidance of their causes; and it is as useless to look for relief in medicines, while the causes are in operation, as to prevent the finger from burning as long as it is in the fire. The cure in the latter case must begin with taking it out of the fire. If there is excessive use of any part of the nervous system, whether of the thinking portions or of the propensities, the remedy is rest; non-indulgence in the first place, and then exercising the thoughts and propensities in another direction, to the extent, if possible, of an almost entire forgetfulness of previous studies and appetites. If, for example, a man has studied himself into a diseased condition; if he has had such weighty responsibilities resting on him, that the draft upon his brain is such that he can not get sufficient sleep, and he either becomes deranged or is on his way to the grave by nervous prostration, there is no more safe and certain means of perfect relief, than that of sending out the nervous influence, or "stores," or accumulations, in a different direction—that, for example, of absorbing and pleasurably interesting out-door activities. For the nervous fluids are constantly being generated, as steam in a locomotive, when the fire is kept burning; and if that steam is not expending itself on the driving mechanism, it must be let out upon the air. Destruction is inevitable, unless it finds vent somewhere. Hence the process of cure for all those nervous maladies which arise from over-use, is not merely a cessation of such uses, which is rest, but an employment of the influences in a different direction; because, without such employment, there is no perfect rest, as from mere force of habit these influences will go out through the accustomed channels. To prevent a country from being devastated by a rising river, not only must a dam be built, but an outlet must be opened in another direction.

A Russian nobleman, childless, was banished to Siberia. His wife was permitted to share his toils and privations. They were compelled to live on the plainest fare, to live in a miserable hut, and work hard every day. At the end of fifteen years, they had a house full of healthy children. In this case, the power of reproduction was lost through those excessive indulgences which are inseparable from a life of idleness and voluptuous ease. Hard-working peoples are the most prolific, as witness the Israelites in the laborious service of Pharaoh. In a recent census taken in one of the towns of Massachusetts, it appeared that although the foreign population was less than the native, a smaller number of Irish families gave more births in one year than a larger number of American. An Irishman does more hard work than an American. Idleness predisposes to an excessive indulgence of the propensities, and this very indulgence increases the desires; thus being over-used, they become powerless, inefficient; that upon which they thus feed inordinately, destroys them. A state of labor is the natural habitual state of man; animal indulgences, incidental, occasional; and in proportion as this law is reversed, in such proportion does it tend to the extinction of the race.

The object of this extended statement, is to impress on the mind that the natural, safe, and efficient means of correcting all nervous derangements, is to give more rest to the parts deranged or disturbed, and so to change the modes of life as to send out the nervous power constantly being generated in the system, through other channels, thus giving those which are overworked time for recuperation. It would be the same if a man were dying with excessive physical exertion. Let the body rest, and give him something to engage his thoughts pleasantly; send the nervous system out of the body, through the brain.

Next to over-indulgences as a cause of nervous disturbances, is an imperfect assimilation of food, that is, indigestion, known as dyspepsia, which is the failure of the stomach and other parts of the digestive apparatus to convert the food into perfect, that is, health-giving blood; for if the blood is imperfect in quality, the nervous influence which is made out of that blood must also be imperfect, not of a suitable character, hence does not manifest itself naturally, fails to effect the objects intended by nature. When a man is dyspeptic, that portion of the nervous fluid which is sent to the brain is not of a proper quality; and whatever part of the brain is in the habit of greater exercise, is more particularly disturbed, because more of the imperfect blood, or more of the imperfect nervous fluid is sent there. Suppose the moral organs, at the top of the head, are most constantly exercised, as in the case of a clergyman, his teachings will diverge from the right line, will be unfaithfully lax, or morbidly rigid, painfully exact, unsympathizing, vituperative, dealing in epithets and invectives, with not a tithe of the forbearances which characterized the Master. If he be more of a theorizer, more purely intellectual or imaginative, his discourses will tend to what is airy, impractical, and absurd. If he be "domestic," a great lover of his wife and children, devoted to their welfare, the effect of bad blood on this part of the brain is to revolutionize this sentiment, and he becomes insufferably cross, complaining of the very things done for his comfort and welfare, overrunning with a multitude of utterly groundless suspicions, imagining slights and inattentions where they were never intended, and perverting every thing said and done. If such a person, or exceedingly affectionate parent, or other relative, becomes actually deranged, the life of the child is sought, or of the kindred most loved. It is thus that mothers are not unfrequently known to murder their own children, the infants of their bosom.

If a man loves to eat over-much, the imperfect, the bad blood excites the stomach to inordinate appetites. The man is never satisfied; he is always eating, always hungry, can not wait for his meals with any kind of comfort or patience—hence eats whenever he is hungry, giving the stomach no time for rest; thus it is over-worked, and the main difficulty is increased.

The practical view to be taken of nervous affections in general, is, that they are an effect; and whether it be called neuralgia, nervous debility, nervous prostration, or any other name, and in whatever part of the body it is located, the immediate cause is in the condition of the blood, for it is upon the blood the nerves feed, it is by the blood they are nourished, and from it they derive all their power. If the blood is not supplied in sufficient quantity, inanition is the result, a general prostration; if the blood is too rich, there is abnormal action; if the blood is impure or imperfect, there is nervous irritability; the mind is fretful, peevish, unstable, the body is weak, restless, and invigorous; if the blood is over-abundant, there are aches and pains, neuralgias, which are literally "nerve-aches," in any and every part of the system. There is beside these, a nervous debility, which arises from the part being exercised beyond the strength given by the natural amount of healthful blood sent to it, and that part becomes exhausted temporarily; if rested, it returns to its natural condition; if called into excessive action soon again, rest will enable it to regain its usual strength; but that rest must be longer, each succeeding exhaustion requiring more time for recuperation, until, eventually, the power of recuperation is lost. This is destructive excess, not only to the part itself, but to the whole system, because the malady spreads as naturally and as certainly as the fire in a burning building, and ceases not until the ruin is complete. If the brain is exercised too intensely, whether in perplexing study, in incessant anxieties, or in the vortex of business, it soon begins at length to lose its elasticity, its power of concentration, its continuity of thought, and the mind goes out in darkness, the body in death, or both body and mind together wilt and wither away. But even this condition of things is found in an unnatural state of the blood, brought about by the brain consuming more than its share of the nervous supplies; hence the stomach and other portions of the digestive apparatus have less than their share, perform their duties imperfectly, and make an imperfect blood, bringing us again to the point arrived at before, to wit, that in the cure of all nervous difficulties, rest to the parts is the first essential; the absolutely indispensable step; the next is to supply the parts with a better quality of blood, a blood which is perfect, pure, and abundant. Nothing can purify the blood without pure air; nothing can make it perfect and life-giving but muscular exercise, sufficient, yet not excessive, not exhausting, the whole expressed in three words, "MODERATE OUT-DOOR ACTIVITIES," always safe, always permanently efficient, and will always cure, if cure is possible.

In addition to these, and without which the others can not be expected to be efficient, the nervous influences must be sent out of the body through another set of channels; must be expended in physical exercises, steady, hard, remunerative work, calling into requisition, the while, all that force of will which can possibly be brought to bear in compelling the mind into a different channel.

The proof of the truthfulness of the principle presented may be easily demonstrated in any half-hour. Move the arm up and down continuously, until motion becomes painful or impossible; then running can be done as vigorously as if the arm had not been moved so. After running for some time, and resting the arm, it recovers its entire strength. It is precisely so with every other muscle or set of muscles in the system, its glands or manufactories. A man may think until the brain seems scarcely to work at all, yet he can go out and work as hard as before he began to think, and after a while can go to his study and think to advantage again.

To administer medicines to stimulate any power into wonted activity, is only the stimulus of the lash to an exhausted donkey; it either kills outright, or induces an unnatural effort, which can only be exerted temporarily, with the certain effect of falling into greater exhaustions. Precisely so is it with the tonics and other remedies more powerful and more destructive, when employed to "invigorate." As proof, the universal testimony is, "It seemed to do good for a while." The recognition of this simple truth would prevent the blasting of many a fond hope, would save many a dollar to those who can ill afford its expenditure, would prevent the robbery of many a till, would save his integrity to many a (heretofore) noble-minded youth. Ignorance of that principle has allowed multitudes to precipitate themselves into wrong-doing, and into vices which have ultimated in ruin to body, soul, and estate.

